# The impact of a diagnostic decision support system on the consultation: perceptions of GPs and patients

**DOI:** 10.1186/s12911-017-0477-6

**Published:** 2017-06-02

**Authors:** Talya Porat, Brendan Delaney, Olga Kostopoulou

**Affiliations:** 1 Department of Primary Care and Public Health Sciences, King’s College London, 3rd floor Addison House, Guy’s Campus, London, SE1 3QD UK; 2 Department of Surgery and Cancer, Imperial College London, London, UK

**Keywords:** Decision support systems, Diagnosis, Diagnostic error, Usability, Usefulness, Acceptability, Patient satisfaction, Electronic health record, Cognitive engineering

## Abstract

**Background:**

Clinical decision support systems (DSS) aimed at supporting diagnosis are not widely used. This is mainly due to usability issues and lack of integration into clinical work and the electronic health record (EHR). In this study we examined the usability and acceptability of a diagnostic DSS prototype integrated with the EHR and in comparison with the EHR alone.

**Methods:**

Thirty-four General Practitioners (GPs) consulted with 6 standardised patients (SPs) using only their EHR system (baseline session); on another day, they consulted with 6 different but matched for difficulty SPs, using the EHR with the integrated DSS prototype (DSS session). GPs were interviewed twice (at the end of each session), and completed the Post-Study System Usability Questionnaire at the end of the DSS session. The SPs completed the Consultation Satisfaction Questionnaire after each consultation.

**Results:**

The majority of GPs (74%) found the DSS useful: it helped them consider more diagnoses and ask more targeted questions. They considered three user interface features to be the most useful: (1) integration with the EHR; (2) suggested diagnoses to consider at the start of the consultation and; (3) the checklist of symptoms and signs in relation to each suggested diagnosis. There were also criticisms: half of the GPs felt that the DSS changed their consultation style, by requiring them to code symptoms and signs while interacting with the patient. SPs sometimes commented that GPs were looking at their computer more than at them; this comment was made more often in the DSS session (15%) than in the baseline session (3%). Nevertheless, SP ratings on the satisfaction questionnaire did not differ between the two sessions.

**Conclusions:**

To use the DSS effectively, GPs would need to adapt their consultation style, so that they code more information during rather than at the end of the consultation. This presents a potential barrier to adoption. Training GPs to use the system in a patient-centred way, as well as improvement of the DSS interface itself, could facilitate coding. To enhance patient acceptability, patients should be informed about the potential of the DSS to improve diagnostic accuracy.

**Electronic supplementary material:**

The online version of this article (doi:10.1186/s12911-017-0477-6) contains supplementary material, which is available to authorized users.

## Background

Computerised clinical decision support systems (DSS) are increasingly important in primary care for providing patient-specific, evidence-based advice for General Practitioners (GPs) [1–3]. GPs in the UK are family physicians with a gatekeeping role, controlling access to specialist services. They deal with a wide range of disease areas and have the difficult task of detecting uncommon but potentially serious diseases among common non-serious complaints.

Despite evidence that DSS improve GPs performance [4–6], their adoption in clinical practice is very limited [7, 8] and includes mainly alerts and reminders designed to support prescribing, treatment and disease management decisions [9, 10]. Measuring performance and quantifiable benefits of a DSS is necessary but having good results on these measures does not necessarily predict adoption in practice [2].

There are several reasons GPs may be reluctant to adopt a DSS [1, 3, 8]. Usability issues including lack of integration into the clinical work is cited as a main barrier to broad adoption [9, 11–13]. This includes lack of integration with the EHR, which is important in order to trigger relevant patient information at appropriate points in the cognitive workflow and to prevent double entry of data, to both the DSS and EHR [14, 15].

Other concerns that specifically relate to diagnostic DSS are perceived need, and perceived challenge to one’s authority as a knowledgeable professional. It may be easier for GPs to acknowledge the need for support of memory-based tasks (e.g., prescribing, screening), rather than judgment-based tasks, such as diagnosis. They may, for example, not believe that their expert judgment can be reduced to a few rules, despite substantial evidence that the “actuarial” method (using a clinical prediction rule or formula that is based on and combines the evidence) performs better than unaided clinical judgment [16].

GPs may also be concerned that their patients and/or colleagues would think less of them, if they used a diagnostic DSS. They may finally think that it will be time-consuming, and detract from the doctor-patient relationship [17]. There is indeed evidence that, in hypothetical clinical scenarios, GPs who do not use decision aids are thought as having higher diagnostic ability than those who do [18, 19] though this difference may be attenuated if the decision aid has been “developed at a prestigious institution” [18]. However, evidence that patients may derogate GPs who use decision aids comes from studies where participants were students reading hypothetical medical scenarios. We reviewed the literature, and to our knowledge, this is the first study that examined patients’ perceptions of GPs using a DSS in a naturalistic environment with standardised patients.

As part of the Translational Medicine and Patient Safety in Europe (TRANSFoRm) project (www.transformproject.eu) we designed, developed and evaluated a diagnostic DSS prototype for use in primary care [6, 20–22].

The DSS prototype was designed to support GPs cognitive requirements in the diagnostic process with the aim of designing a usable system that will integrate with the GPs’ clinical work [22]. We employed cognitive engineering methods [23, 24] to identify key decisions and uncover decision requirements in the diagnostic process. The decision requirements then guided the design of the system, specifically aiming to help GPs generate more diagnostic hypotheses to reduce narrow focus on one diagnosis developing early in the clinical encounter, and remind GPs of the key questions that they need to ask. Key features of the tool are described in Table 1. The evaluation of the prototype in a high-fidelity simulation found it to improve diagnostic accuracy and management without increasing consultation time and investigations ordered [6]. In this paper we report findings about the users’ perceived usability and acceptability of the DSS, and the patients’ satisfaction from the consultation, as measured during the evaluation study using interviews and standardised questionnaires. We aimed to identify facilitators and barriers to future DSS adoption.

**Table 1:** Key features of the diagnostic DSS prototype

• The DSS is integrated with Vision EHR system (www.inps4.co.uk/vision) and is triggered by the GP entering the reason for encounter (RfE).
• Upon being triggered, the DSS presents a list of possible diagnoses, based on the RfE and information in the patient’s record (age, sex and risk factors). The intention is for the list to appear as early as possible, before GPs start gathering any further information.
• The interface allows the GP to easily code the patient’s signs, symptoms and examinations using a context-sensitive search box, while the order of diagnoses on the list is updated according to user input.
• For each suggested diagnosis, the GP can view a checklist of associated symptoms and signs, and indicate their presence or absence.
• At the end of the consultation, all the information is transferred automatically to the patient’s electronic health record, with the correct codes and structure.

Further information and screen examples are provided in [6]

## Method

### Design and procedure

A detailed description of the DSS evaluation study is provided elsewhere [6]. To summarise, 34 GPs who were using Vision EHR at their practice diagnosed 12 standardised patients (SPs) in simulated consultations at King’s College London. Each GP first consulted with 6 SPs using only their usual EHR system - Vision (‘baseline session’), and on another day, with 6 different but matched for difficulty SPs, using the EHR with the integrated DSS prototype (‘DSS session’). Before the DSS session, participants were introduced to the DSS prototype and its functionality, and performed training scenarios with the DSS (total training time was 20–30 min). GPs were interviewed after the baseline and the DSS sessions.

The first question enabled participants to comment about the session in general:

- *“Do you have any comments about today’s session? Feel free to comment on anything you want”.*

At the end of the baseline session, GPs were asked about the likely usefulness of a diagnostic decision support tool in clinical practice:

- *“Do you think that a computerised diagnostic support tool, integrated with the patient EHR, would have helped with any of the patients today? Which patients?”*

At the end of the DSS session, GPs were interviewed about their experience using the DSS prototype, and were asked whether it had helped them:

- *“Do you think that the diagnostic support tool helped with any of the patients today? Which patients?”*

GPs were also asked if they had suggestions for improving the tool and the data collection process. GPs then completed the Post-Study System Usability Questionnaire (PSSUQ). The IBM PSSUQ [25] consists of 19 questions, answered on 7-point Likert scales, from 1 “strongly disagree” to 7 “strongly agree”, allowing for a “not applicable” option. The PSSUQ evaluates 4 dimensions: overall satisfaction; system usefulness; information quality and interface quality (see Additional file 1). It is accepted as a valid and reliable instrument, and it is used frequently in research [26].

At the end of each consultation, after leaving the room, the SPs completed a standardized Consultation Satisfaction Questionnaire (CSQ) [27]. The CSQ is a validated and reliable questionnaire [28–30] consists of 18 questions in which participants respond to statements about how they felt about the consultation on a 5-point Likert scales from ‘strongly agree’ to ‘strongly disagree’. The CSQ evaluates 4 dimensions: general satisfaction; professional care; depth of relationship and length of consultation (Additional file 2).

### Analysis

A thematic analysis approach was used to identify themes and subthemes [31] related to the usability of the DSS and suggestions for improving the tool.

The qualitative data from interviews and questionnaires were transcribed in full, stored, coded and analysed using NVivo Version 10 software. Out of the 68 interviews (34 GPs being interviewed twice – after the baseline and after the DSS sessions) and 34 completed questionnaires (PSSUQ), 10 interviews (5 after the baseline session and 5 after the DSS session) and 5 questionnaires were pilot coded by TP and OK separately to develop an initial coding framework. Where there were differences these were discussed to resolve them until consensus about the coding was reached. Based on the coding framework, TP coded the rest of the interviews and questionnaires. The final coding was then reviewed and discussed by all authors, and minor changes to the coding were made. Statistical analyses of the questionnaire data are reported by [6].

## Results

Thirty-four GPs from Greater London using Vision (www.inps4.co.uk/vision) EHR system participated in the study (17 males). The average number of years practicing family medicine was 12.6 (SD 12.57, range 1 month to 40 years) and the average number of years using the Vision EHR system was 7.2 (SD 5.6, range 5 months to 17 years).

### Perceptions of GPs

Thematic analysis resulted in 3 major themes: perceived usefulness of the DSS, impact of the DSS on the consultation, and suggestions for improving the DSS. These are described below.

#### Theme 1. Perceived usefulness of the DSS

Perceived usefulness relates to the degree to which users believe that using the technology will help them improve their work and problem solving performance [32, 33]. We identified three sub-themes that relate to GPs’ perceived usefulness of the DSS prototype: supporting GPs in the diagnostic process, useful user interface features and perceived usability.

##### Supporting GPs in the diagnostic process

At the end of the baseline session, when participants were asked whether a diagnostic support system could have helped them with the patients they had just seen, 8 (23.5%) gave positive responses, 17 (50%) gave neutral answers (do not know, it depends), while 9 participants (26.5%) did not think that such a system could have helped.

After using the diagnostic support system, at the end of the DSS session, participants were asked if the DSS had helped them diagnose, and which patients it had helped them with. We categorised their responses into ‘always helpful’, ‘sometimes helpful’, ‘unsure’ and ‘not helpful’. Eight GPs (23.5%) found the diagnostic tool helpful in all the cases and seventeen GPs (50%) found the tool helpful in some of the cases - mainly those considered to be complex:

“[The tool helped] in all of them to an extent. The prompts were useful, organising your own mind. In general, it widened up more things to consider.” (GP 24)

“Yes, the tool helps in elaborating the symptoms more thoroughly, you analyse the different symptoms.” (GP 22)

“I think in straightforward cases it doesn’t really help, like in the UTI… Only when uncertainty increases [it helps]. In the last case [colorectal cancer] it helped. I used it as a checklist, could it be cancer?” (GP3)

Four GPs (11.8%) said that they were unsure if it helped them and five (14.7%) said that the DSS did not help them:

“I personally don’t think tools help me too much, interfere with my process of thought, would have done better without it, it was unnatural.” (GP 5)

“It hindered me, it slowed me down. I already had my working diagnosis in mind. The system had 20 items, I had about 5 in my head.” (GP 15)

“[The DSS] didn’t help but did not distract me from the way I thought.” (GP 14)

GPs elaborated on how the diagnostic tool had helped them: 16 (47%) said that it reminded them to ask important questions that otherwise they might have forgotten, and to ask more targeted questions; 10 GPs (29%) said that it helped them consider more differentials, especially less common ones.

“Reminded me to ask questions. Served as a prompt - reminded me to ask about blood in stool which I would have forgotten.” (GP6)

“You start to think about the unusual things more, compared to without the system. You ask more questions. You start wide and then focus on it, sometimes you need to open up again.” (GP3)

“Helpful to refresh your memory about things that are rare…we mainly stick to the phrase: ‘common things are common’.” (GP7)

“I think it widens your diagnosis, like the guy that had COPD and then ended up with AS. I would get to the diagnosis, but it would be longer without the tool.” (GP16)

Four participants (12%) claimed that they felt more uncertain and reflective with the tool:

“I am more reflective with this: have I missed something here?” (GP8)

“You ask more questions, more uncertainty even if you were certain.” (GP3)

We note that certainty about the diagnosis, measured on a 0–10 VAS, significantly improved when the DSS was used [6].

##### Useful user interface features

GPs explicitly mentioned the following UI features to be most useful:

- Integration with Vision – nine GPs (26%) mentioned the advantage of integrating the DSS with Vision, indicating that it is well integrated and/or could be better integrated (see suggestions for improvements).
  “Useful, good to have it in Vision, you can access it quickly.” (GP 10)
  “It integrates very well with Vision.” (GP6)
  “[The tool] should be integrated better with Vision, should be a part of Vision like an added box [part of Vision main screen].” (GP 35)
- Initial list of suggested diagnoses – four GPs indicated that the initial list was useful.
  “The first bit when you put the reason for encounter and receive the list is a good idea.” (GP14)
  “I did get used to it and it had benefits, especially the list in the beginning, makes you think.” (GP25)
- Symptoms associated with each diagnosis – eight GPs (23.5%) found useful the option to click on a suggested diagnosis, view its associated symptoms and signs and indicate their presence or absence.
  “There were a lot of questions I wrote in free-text but the system didn’t get it. I then ticked the checkboxes under the diagnosis - which was very useful.” (GP33)
  “It was really easy and efficient clicking in the list and asking the relevant symptoms.” (GP7)
  “It was helpful in one of the cases actually, a symptom I forgot to ask. In the last case also – ‘being comfortable when lying flat’ - even if it was negative.” (GP 17)
  Most GPs (31/34, 91%) clicked on a suggested diagnosis to view the associated symptoms and signs at least once in the DSS session.

##### Perceived usability

Table 2 presents mean agreement with each of the 19 statements of the PSSUQ (the post-study system usability questionnaire). Ratings were provided on 7-point Likert scales from 1 (strongly disagree) to 7 (strongly agree), with higher ratings indicating higher satisfaction. The DSS scored well, with average ratings on most questions above 4 (the scale midpoint). GPs were satisfied with how easy (Q1) and simple (Q2) the tool was to use and learn (Q7); the information provided was easy to understand (Q13), clear (Q11) and well organised (Q15); they liked using the interface (Q17) and thought that it was pleasant (Q16). However, the average ratings on Q4 (timely task completion) and Q9 (informative error messages) were below 4, suggesting low satisfaction. GPs’ responses to Q9 were about program bugs, as the interface did not provide any error messages.

**Table 2:** Post-study system usability questionnaire (PSSUQ) results

PSSUQ question	Mean (SD)
Q1 Overall, I am satisfied with how easy it is to use this system.	4.85 (1.42)
Q2 It was simple to use this system.	4.88 (1.63)
Q3 I could effectively complete the tasks and scenarios using this system.	4.47 (1.62)
Q4 I was able to complete the tasks and scenarios quickly using this system.	3.82 (1.31)
Q5 I was able to efficiently complete the tasks and scenarios using this system.	4.41 (1.42)
Q6 I felt comfortable using this system.	4.53 (1.58)
Q7 It was easy to learn to use this system.	5.47 (1.38)
Q8 I believe I could become productive quickly using this system.	4.27 (1.68)
Q9 The system gave error messages that clearly told me how to fix problems.	3.26 (1.73)
Q10 Whenever I made a mistake using the system, I could recover easily and quickly.	4.39 (1.67)
Q11 The information (such as on-line help, on-screenmessages and other documentation) provided with this system was clear.	4.95 (1.59)
Q12 It was easy to find the information I needed.	4.69 (1.55)
Q13 The information provided for the system was easy to understand.	5.22 (1.34)
Q14 The information was effective in helping me complete the tasks and scenarios.	4.53 (1.34)
Q15 The organization of information on the system screens was clear.	4.88 (1.62)
Q16 The interface of this system was pleasant.	5.21 (1.39)
Q17 I liked using the interface of this system.	4.88 (1.49)
Q18 This system has all the functions and capabilities I expect it to have.	4.09 (1.59)
Q19 Overall, I am satisfied with this system.	4.18 (1.55)

#### Theme 2: Impact of the DSS on the consultation

We identified two sub-themes relating to the impact of the DSS on the consultation: impact on consultation style and GP-patient interaction, and time concerns.

##### Impact on consultation style and GP-patient interaction

During the baseline session, 73.5% of the participants recorded information only at the end of the consultation. It is thus not surprising that half of the participants felt that the diagnostic tool, which required data entry during the consultation, influenced their consultation style:

“It’s completely different to how I normally work, unsettling and confusing, I’m not quite sure where I am, to what extent I’m following a template and how I take history.” (GP8)

“You need to get used to it…I do my consultations in a different way, but it works quite quick.” (GP12)

“It felt different, I definitely used different questions. From a doctor’s point of view it’s good, from the patient’s point of view it’s weird…I would have asked more open questions relative to closed ones.” (GP16)

“It throws my standard history examination style, stop me from doing unnecessary investigations.” (GP 9)

Eight GPs (23%) were concerned that typing into the computer during the consultation would interfere with the doctor-patient interaction and communication, because it might reduce eye contact with the patient:

“I normally chat and look at the patients, it throws my normal thing.” (GP9)

“I usually don’t code during the consultation, less contact with patient, my style is to listen for a long time.” (GP3)

##### Time concerns

Thirteen GPs (38%) felt that the consultation took longer with the tool than without, mainly because they had to search for and select the right code for each symptom (from a drop-down menu based on predictive text from the underlying knowledge base). Using only the EHR, GPs wrote mainly free text, which they perceived to be faster:

“Using the tool in the present format is time consuming, need to speed it up.” (GP7)

“It will be hard to use it in a 10-min. consultation.” (GP22)

“It was too time consuming selecting all the symptoms, you need to minimise interaction during consultation, I would group relevant symptoms together.” (GP13)

Across GPs, the average consultation time did not significantly differ between baseline and DSS sessions [6]. The 13 GPs who expressed concerns about time took slightly longer when using the DSS (mean time 15.45 min) than in the baseline session (mean time 13.53 min), paired samples t-test: 2.13, df = 12, *p* = 0.055.

Despite GPs’ concerns about time, they believed that they could become better using the DSS (see Table 2, Q8 mean rating 4.27) and nine GPs (26%) explicitly mentioned that they got used to the DSS and improved with time.

“Obviously familiarity over time will improve.” (GP19)

“I can see its use. It’s a new thing, with any new system the initial use will affect the customer relationship. As it was going on, it was getting better.” (GP26)

“I did get used to it and it had benefits.” (GP25)

#### Theme 3: Suggestions for improving the DSS

Participants suggested a number of ways, which they believed could improve the DSS:

1. Advanced technology, such as predictive texting, to enable a more natural way of entering data, whether coded or free text.
  “One comment box, write everything you want, using a smart technology, the system could read everything you wrote and come up with a diagnosis on its own.” (GP 15)
  “Why can’t the system analyse free text?” (GP 6).
2. Additional functionalities, such as adding investigations (e.g., X-rays, blood tests, ECG, etc.) to the relevant symptom/sign list for each suggested diagnosis; alerting the GP if the patient came for the same reason for encounter in the last 6 months.
  “Advising what tests one should do; if for example there is blood in sputum - suggest chest x-ray - possible investigations.” (GP 11)
  “If the patient came for the same reason in the last 6 months - indicate it in the system.” (GP 6)
3. Complete integration with EHR systems, including integration with the latest National Institute for Health and Care Excellence (NICE) guidelines and QoF (Quality and Outcomes Framework). For example, after selecting a diagnosis, provide the most updated guidelines describing the appropriate next steps.
  “After you select a diagnosis, for example, asthma the system should ask have you done this, a table at the end, for example, saturation, BP, Pulse and what is missing. What legally I have to do in Asthma.” (GP 9)
  “[The DSS] should integrate with QoF data, for example, COPD performance management domain instead of entering again [the data] to the QoF
  system.” (GP 13)
4. Changes to the interface design. Such suggestions included: highlighting serious diagnoses on the initial list; displaying the precise likelihood of each suggested diagnosis; reducing the length of the suggested diagnoses list; and grouping examination results together to facilitate coding of findings (e.g., selecting ‘abdominal examination’ could display relevant attributes such as: tenderness, guarding, rebound, etc. and the user would select if the attribute was present or absent. The same can be done for basic examination, such as: temperature, blood pressure, pulse).
  “Group examinations, for example respiratory symptoms, same for abdominal. abdo pain – yes/no, if yes then mass/guarding/PR examination and then the diagnoses on the right will change and display.” (GP 33)
  “Add prevalence, probabilities. The tool didn’t help in that, not enough to flash - it can be cancer, but add probabilities, it could be cancer above 30%. Move them up the list and make you do something.” (GP 29)

### Patients satisfaction with the consultation

The standardised patients (SPs) filled in the CSQ questionnaire at the end of each consultation and wrote free-text comments. Satisfaction did not differ between the baseline and DSS sessions, on average and in any of the CSQ dimensions: professional care, depth of the doctor-patient relationship, length of consultation and general satisfaction [6].

All comments were analysed thematically [31] and divided broadly into comments about GP characteristics (e.g., “very kind and professional doctor”), and comments about the use of the computer (e.g., “he was constantly looking at the computer”).

SPs made comments about the GP looking at the computer in six out of 204 consultations at baseline (3%), and in 30 out of 204 consultations (15%) in the DSS session:

“There were times when the doctor was talking to me, not needing to check anything in the computer, but still looking at it.” (SP2)

“The technology was in the way of his communication with me.” (SP1)

The SPs did not make any comments about the DSS specifically or about changes to the GP’s consultation style and way of questioning when using the DSS.

## Discussion

It is encouraging that GPs recognised the usefulness of the DSS. Perceived usefulness is an important facilitator and driver for adoption [8]. Before experiencing its use, the majority of GPs were ‘agnostic’ about its usefulness (77%). This is in line with a recent Nesta report examining GPs’ responses on a survey about adopting innovations in practice [34]: almost 40% said that they would like to adopt IT innovations, but only 7% said that they would like to adopt diagnostic technologies. We found that after using our DSS, the majority of the GPs found it useful, because it helped them consider more diagnoses and ask more targeted questions (74%).

GPs identified three user interface features as helpful in the diagnostic process: (1) the EHR integration; (2) the initial patient-tailored list of potential diagnoses and; (3) the option to expand a diagnosis to its list of relevant symptoms and signs. When designing the DSS, we considered the first feature necessary for ease of use and future adoption. This feature is critical for almost any DSS to trigger relevant patient information at appropriate points in the physician’s workflow. For example, as part of the ESPRC CONSULT project (EP/P010105/1), we are integrating a DSS with the EHR to promote holistic care in stroke patients. The second feature was designed and tested extensively in studies with large samples of GPs in two European countries [20, 21]. It is the most defining feature of the DSS, which sets it apart from other existing diagnostic support systems. The third feature was elicited during an extensive user and decision requirements elicitation process that involved multiple analytical methods and data sources [22].

As reported elsewhere, the DSS did not influence perceptions of the GPs’ professionalism and care, or the general patient satisfaction from the consultation [6]. There was some indication that GPs tended to look at their computer more when using the DSS: the SPs commented about it more often when the DSS was used than when it was not. Nevertheless, such comments were only made in 15% of the consultations, where the DSS was used. We should also take into account that this was the first time that the GPs used the DSS, and after a brief training. Longitudinal studies have shown that the impact of health information technologies on physician-patient interactions (e.g., patient satisfaction, communication about medical issues) improves significantly after using the technology for a period of time [35]. More extensive training and practice with the DSS, and an improved interface design can result in a more seamless integration with the consultation. Furthermore, educating patients about the benefits of the DSS is likely to enhance patient acceptance.

We recognise that a significant barrier for the adoption of the DSS would be the change to many GPs’ style of consultation and documentation, which would be required for the effective use of the DSS. This involves the coding of individual symptoms and signs during the consultation, so that the order of suggested diagnoses is updated according to the evidence accumulated. When using their usual EHR (Vision), most study participants (73.5%) recorded patient information only at the end of the consultation [6]. To the extent that this is representative of how GPs document the clinical consultation, and it is not specific to Vision, it can present substantial challenges to adoption. Pitted against this challenge are the GPs’ perceived usefulness of the DSS, and the lack of any measurable influence on patient satisfaction from the consultation when GPs coded during the consultation (when using the DSS). Furthermore, the dynamic updating of the diagnostic list as information is coded into the EHR, provides an incentive for GPs to code information during the consultation – currently, they do not have to code.

A recent report identified time constraints as the main barrier for the adoption of innovations by GPs [34]. In our DSS evaluation study, a substantial minority of GPs (38%) found using the DSS time consuming. Although the GPs who expressed concerns about time took slightly longer when using the DSS, this was not the case for the GP sample as a whole. Reducing the time the GP interacts with the computer can be achieved in a number of ways, for example, by improving the integration of the DSS with the EHR and other related systems; grouping examination results together in order to reduce the number of clicks required to enter information; and finding more natural ways to record data such as predictive texting and voice to text. Another solution could involve patients entering their data prior to the consultation, which could also enhance a shared understanding of the health record.

We evaluated the DSS prototype in a high-fidelity simulation with 34 GPs, while most system evaluations are performed with 5 to 10 users [36]. This significant amount of users enabled us to report quantitative results [6] in addition to the qualitative findings.

To maintain some control over the study design, we employed actors rather than real patients. These actors are specifically trained to depict patients, in order to assess clinical communication skills and quality of care, and are used to rating satisfaction from the consultation (e.g., [37]). Extensive medical-education literature describes the successful use of SPs and reports that SPs can capture variation in clinical practice [38–40].

Nevertheless, the use of actors rather than real patients is a limitation, as there may be a concern that, since they are not experiencing the specific health complaint, their assessments may not be a valid reflection of patient perceptions. Actors however have been patients in their own right and have consulted the GP; they have expectations from the consultation which would influence their assessments. Real patients would still provide their own perspective, influenced by their own experiences and expectations, and would not represent the whole range of patient expectations. This is the first study that examines patients’ perceptions of GPs using a DSS in a naturalistic environment, during a simulated consultation with standardised patients. Previous research employed students as participants, dealing with hypothetical scenarios [e.g., 18,19]. While hypothetical scenarios (vignettes) appear to be a valid and comprehensive method to measure physicians’ quality of care [41], they may have limitations in external validity [18] compare to a naturalistic environment using SPs. In addition, SPs are likely to have more experience in interactions with physicians than students [19].

## Conclusions

GPs reported that the DSS helped them in the diagnostic process, specifically in considering more diagnoses and asking more targeted questions. However, to use the DSS effectively, GPs would need to adapt their consultation style, so that they code more information during rather than at the end of the consultation. This could be a potential barrier to adoption. Training GPs to use the system in a patient-centred way, as well as improvement of the DSS interface itself, could facilitate coding. To enhance patient acceptability and satisfaction, patients should be informed about the potential of the DSS to improve diagnostic accuracy. The feasibility and acceptability of engaging the patient with the DSS before, during and after the consultation could also be explored. Future work includes updating the DSS prototype based on the feedback we received from the GPs, and evaluating its usability and acceptability in real practice.

## Additional files

Additional file 1: Post-Study System Usability Questionnaire (PSSUQ). Consists of 19 questions, answered on 7-point Likert scales (from 1 “strongly disagree” to 7 “strongly agree”). (PDF 62 kb)

Additional file 2: Consultation Satisfaction Questionnaire (CSQ). Consists of 18 questions, answered on 5-point Likert scales (from “strongly agree” to “strongly disagree”). (PDF 57 kb)

## Abbreviations

CSQ: Consultation Satisfaction Questionnaire
DSS: Decision support system
EHR: Electronic health records
GP: General Practitioner
PSSUQ: Post-Study System Usability Questionnaire
RfE: Reason for encounter
SP (s): Standardised patient (s)
