# Supplementary material for: The impact of a diagnostic decision support system on the consultation: perceptions of GPs and patients
Source: BMC Med Inform Decis Mak. 2017 Jun 2;17:79. doi: 10.1186/s12911-017-0477-6 (PMC5457602; doi:10.1186/s12911-017-0477-6)
Supplement: Supplementary file 1 — Post-Study System Usability Questionnaire (PSSUQ). Consists of 19 questions, answered on 7-point Likert scales (from 1 “strongly disagree” to 7 “strongly agree”). (PDF 62 kb) [file 12911_2017_477_MOESM1_ESM.pdf]

Post-Study System Usability Questionnaire (PSSUQ)

1. Overall, I am satisfied with how easy it is to use this system.

N/A **STRONGLY DISAGREE** **1 2 3 4 5 6 7** **STRONGLY AGREE**

COMMENTS:

2. It was simple to use this system.

N/A **STRONGLY DISAGREE** **1 2 3 4 5 6 7** **STRONGLY AGREE**

COMMENTS:

3. I could effectively complete the tasks and scenarios using this system.

N/A **STRONGLY DISAGREE** **1 2 3 4 5 6 7** **STRONGLY AGREE**

COMMENTS:

4. I was able to complete the tasks and scenarios quickly using this system.

N/A **STRONGLY DISAGREE** **1 2 3 4 5 6 7** **STRONGLY AGREE**

COMMENTS:

5. I was able to efficiently complete the tasks and scenarios using this system.

N/A **STRONGLY DISAGREE** **1 2 3 4 5 6 7** **STRONGLY AGREE**

COMMENTS:

6. I felt comfortable using this system.

N/A **STRONGLY DISAGREE** **1 2 3 4 5 6 7** **STRONGLY AGREE**

COMMENTS:

7. It was easy to learn to use this system.

N/A **STRONGLY DISAGREE** **1 2 3 4 5 6 7** **STRONGLY AGREE**

COMMENTS:

8. I believe I could become productive quickly using this system.

N/A **STRONGLY DISAGREE** **1 2 3 4 5 6 7** **STRONGLY AGREE**

COMMENTS:

9. The system gave error messages that clearly told me how to fix problems.

N/A **STRONGLY DISAGREE** **1 2 3 4 5 6 7** **STRONGLY AGREE**

COMMENTS:

10. Whenever I made a mistake using the system, I could recover easily and quickly.

N/A **STRONGLY DISAGREE** **1 2 3 4 5 6 7** **STRONGLY AGREE**

COMMENTS:

11. The information (such as on-line help, on-screen messages and other documentation) provided with this system was clear.

N/A	**STRONGLY DISAGREE**	**1**	**2**	**3**	**4**	**5**	**6**	**7**	**STRONGLY AGREE**

COMMENTS:

12. It was easy to find the information I needed.

N/A	**STRONGLY DISAGREE**	**1**	**2**	**3**	**4**	**5**	**6**	**7**	**STRONGLY AGREE**

COMMENTS:

13. The information provided for the system was easy to understand.

N/A	**STRONGLY DISAGREE**	**1**	**2**	**3**	**4**	**5**	**6**	**7**	**STRONGLY AGREE**

COMMENTS:

14. The information was effective in helping me complete the tasks and scenarios.

N/A	**STRONGLY DISAGREE**	**1**	**2**	**3**	**4**	**5**	**6**	**7**	**STRONGLY AGREE**

COMMENTS:

15. The organization of information on the system screens was clear.

N/A	**STRONGLY DISAGREE**	**1**	**2**	**3**	**4**	**5**	**6**	**7**	**STRONGLY AGREE**

COMMENTS:

16. The interface of this system was pleasant.
Note: *The interface includes those items that you use to interact with the system. For example, some components of the interface are the keyboard, the mouse, the screens (including their use of graphics and language).*

N/A	**STRONGLY DISAGREE**	**1**	**2**	**3**	**4**	**5**	**6**	**7**	**STRONGLY AGREE**

COMMENTS:

17. I liked using the interface of this system.

N/A	**STRONGLY DISAGREE**	**1**	**2**	**3**	**4**	**5**	**6**	**7**	**STRONGLY AGREE**

COMMENTS:

18. This system has all the functions and capabilities I expect it to have.

N/A	**STRONGLY DISAGREE**	**1**	**2**	**3**	**4**	**5**	**6**	**7**	**STRONGLY AGREE**

COMMENTS:

19. Overall, I am satisfied with this system.

N/A	**STRONGLY DISAGREE**	**1**	**2**	**3**	**4**	**5**	**6**	**7**	**STRONGLY AGREE**

COMMENTS:
