# Supplementary material for: The impact of a diagnostic decision support system on the consultation: perceptions of GPs and patients
Source: BMC Med Inform Decis Mak. 2017 Jun 2;17:79. doi: 10.1186/s12911-017-0477-6 (PMC5457602; doi:10.1186/s12911-017-0477-6)
Supplement: Supplementary file 2 — Consultation Satisfaction Questionnaire (CSQ). Consists of 18 questions, answered on 5-point Likert scales (from “strongly agree” to “strongly disagree”). (PDF 57 kb) [file 12911_2017_477_MOESM2_ESM.pdf]

## Consultation Satisfaction Questionnaire (CSQ)

Date: ______________________

Patient name: ________________

GP ID: ___________________

**<u>Consultation Satisfaction Questionnaire</u>**

This form contains a list of questions that ask you what you think of your visit to the doctor today. Your answers will be kept entirely confidential and will not be shown to the doctor so feel free to say what you wish.

Please answer all the questions by placing a tick in the answer box that is closest to what you think. “Neutral” means you have no feeling either way.

| | **Professional care** | **Strongly agree** | **Agree** | **Neutral** | **Disagree** | **Strongly disagree** |
|---|---|---|---|---|---|---|
| 1. | I am totally satisfied with my visit to this doctor | | | | | |
| 2. | This doctor was very careful to check everything when examining me | | | | | |
| 3. | I will follow this doctor's advice because I think he/she is absolutely right | | | | | |
| 4. | I felt able to tell this doctor about very personal things | | | | | |
| 5. | The time I was able to spend with the doctor was a bit too short. | | | | | |
| 6. | This doctor told me everything about my treatment | | | | | |
| 7. | Some things about my consultation with the doctor could have been better | | | | | |
| 8. | There are some things this doctor does not know about me | | | | | |
| 9. | This doctor examined me very thoroughly | | | | | |
| 10. | I thought this doctor took notice of me as a person | | | | | |
| 11. | The time I was allowed to spend with the doctor was not long enough to deal with everything I wanted | | | | | |
| 12. | I understand my illness much better after seeing this doctor | | | | | |
| 13. | This doctor was interested in me as a person not just my illness | | | | | |
| 14. | This doctor knows all about me | | | | | |
| 15. | I felt this doctor really knew what I was thinking | | | | | |
| 16. | I wish it had been possible to spend a little longer with the doctor | | | | | |
| 17. | I am not completely satisfied with my visit to the doctor | | | | | |
| 18. | I would find it difficult to tell this doctor about some private things | | | | | |

Do you have any other comments about the consultation?
